# LY6G6D is a selectively expressed colorectal cancer antigen that can be used for targeting a therapeutic T-cell response by a T-cell engager

**DOI:** 10.3389/fimmu.2022.1008764

**Published:** 2022-09-08

**Authors:** Leticia Corrales, Susanne Hipp, Katharina Martin, Nicolas Sabarth, Iñigo Tirapu, Klaus Fuchs, Barbara Thaler, Christian Walterskirchen, Kathrin Bauer, Markus Fabits, Michael Bergmann, Carina Binder, Paolo ML. Chetta, Anne B. Vogt, Paul J. Adam

**Affiliations:** ^1^ Boehringer Ingelheim Regional Center Vienna (RCV), GmbH & Co KG., Cancer Immunology & Immune Modulation, Vienna, Austria; ^2^ Boehringer Ingelheim Pharmaceuticals, Inc., Translational Medicine and Clinical Pharmacology, Ridgefield, CT, United States; ^3^ Boehringer Ingelheim Regional Center Vienna (RCV) GmbH & Co KG., Biotherapeutics Discovery, Vienna, Austria; ^4^ Boehringer Ingelheim Pharma, GmbH & Co KG, Biotherapeutics Discovery, Biberach, Germany; ^5^ Division of Visceral Surgery, Department of General Surgery and Comprehensive Cancer Center, Medical University of Vienna, Vienna, Austria; ^6^ Department of Pathology, Medical University of Vienna, Vienna, Austria; ^7^ Boehringer Ingelheim RCV, GmbH & Co KG., Oncology Translational Science, Vienna, Austria

**Keywords:** LY6G6D, CD3, TcE (T cell engager), CRC (colorectal cancer), immunotherapy

## Abstract

Colorectal cancer (CRC) is one of the most common cancers worldwide and demands more effective treatments. We sought to identify tumor selective CRC antigens and their therapeutic potential for cytotoxic T-cell targeting by transcriptomic and immunohistochemical analysis. LY6G6D was identified as a tumor selectively expressed CRC antigen, mainly in the microsatellite stable (MSS) subtype. A specific anti LY6G6D/CD3 T cell engager (TcE) was generated and demonstrated potent tumor cell killing and T cell activation *in vitro*. Ex vivo treatment of primary patient-derived CRC tumor slice cultures with the LY6G6D/CD3 TcE led to IFNγ secretion in LY6G6D positive tumor samples. *In vivo*, LY6G6D/CD3 TcE monotherapy demonstrated tumor regressions in pre-clinical mouse models of engrafted human CRC tumor cells and PBMCs. Lastly, 2D and 3D cocultures of LY6G6D positive and negative cells were used to explore the bystander killing of LY6G6D negative cells after specific activation of T cells by LY6G6D positive cells. LY6G6D/CD3 TcE treatment was shown to lyse target negative cells in the vicinity of target positive cells through a combined effect of IFNγ, TNFα and Fas/FasL. In summary, LY6G6D was identified as a selectively expressed CRC antigen that can be utilized to potently re-direct and activate cytotoxic T-cells to lyse LY6G6D expressing CRC using a TcE. This effect can be spread to target negative neighboring tumor cells, potentially leading to improved therapeutic efficacy.

## Introduction

Colorectal cancer (CRC) is the second leading cause of cancer death worldwide and third most diagnosed cancer (1). CRC is a heterogeneous disease composed by different subtypes characterized by genetic and epigenetic alterations of genes that encode mismatch repair (MMR) enzymes (2). Microsatellite instability (MSI) is used as a marker for deficient-MMR (dMMR) and proficient-MMR (pMMR) tumors. Approximately 15% of CRC show an MSI-High phenotype because of DNA mismatch repair deficiency, while 85% of CRC show a MSS or MSI-Low phenotype characterized by a low tumor mutational burden (3). Due to the heterogeneous genetic alterations in CRC, there is a diverse responsiveness of the different CRC subtypes to conventional chemotherapy, targeted therapies, and immunotherapy (4). Due to a higher number of potential mutation-associated neoantigens in dMMR tumors, anti-PD1 antibodies had only shown efficacy in patients with MSI tumors (5). Therefore, there is a need for effective immunotherapies for CRC tumors that do not respond to approved immunotherapies.

The Lymphocyte Antigen 6 Family Member G6D (LY6G6D) belongs to a cluster of leukocyte antigens located in the major histocompatibility complex (MHC) class III region on chromosome 6 (6). LY6G6D, like most member of the family, is attached on the cell membrane by a glycosylphosphatidylinositol (GPI) anchor. LY6G6D was found to be expressed in CRC, especially in the MSS subtype, by transcriptomic analysis (7). Despite of limited knowledge about its function, it has been suggested that it might have a role in regulating tumor growth and immune evasion in CRC (7). LY6G6D specific expression on MSS CRC makes it an interesting target for antibody-based therapies.

Bispecific T-cell engagers (TcE) have become a promising class of antibody-based immunotherapy. Their therapeutic efficacy is well established in hematologic malignancies (8) (9), and more recently a reality for solid tumors after the recent approval of Tebentafusp for uveal melanoma (10). TcE are engineered to bind simultaneously to a tumor antigen and to CD3 on T cells and only when both are engaged it can induce T cell activation by cross-linking of the TCR. Activated T cells produce perforin and granzyme B, leading to cytolysis of tumor cells. In addition, T cells proliferate, and release immune cytokines and chemokines. The activation of T cells within the tumor microenvironment (TME) thus induce inflammation and help turning the tumor into an inflamed phenotype (11–13). Due to their potent mechanism of action, the selection of tumor specific antigens, with no or scarce expression in normal tissues is of central importance to ensure a therapeutic window and prevent TcE-related toxicities.

In this report, we describe the identification of LY6G6D as a tumor selectively expressed antigen in CRC, and the generation of a LY6G6D/CD3 bi-specific antibody to potently redirect T cells into LY6G6D expressing CRC cells. The LY6G6D/CD3 TcE shows specific killing of target positive cells in *in vitro* assays where the expression of LY6G6D is homogeneous among cells. But it also demonstrates lysis of LY6G6D non-expressing cells in cocultures with LY6G6D expressing cells. Mechanistically this bystander killing of LY6G6D non-expressing cells is mediated by Fas/FasL, TNFα and IFNγ. Interestingly, these pathways seem to be dispensable for the direct killing of LY6G6D expressing cells upon engagement by the TcE. Since LY6G6D expression in tumors shows some level of heterogeneity, this finding implies that bystander killing of target negative cells in the tumor could increase the therapeutic effect of the TcE.

## Material and methods

### Human PBMCs and CRC tissues

Frozen human PBMCs were obtained from StemCell Technologies. Human tissues were obtained from the Department of Pathology at the Medical University of Vienna, Austria or Indivumed GmbH, Germany. All blood donors and patients provided written informed consent before sample collection.

### Cell lines

The human CRC cell lines used in this study were NCI-H508, LS1034, SK-CO1, obtained from ATCC; CL-14 from Leibniz Institute DSMZ: and HT55 from Public Health England and NCM460 from Incell. Human kidney cells 293 were obtained from ATCC and 293 cells expressing LY6G6D and Jurkat WT-NFATluc reporter cells were established in-house.

All cell lines were cultured according to supplier’s instruction and maintained at 37°C, 5% CO_2_.

NCI-H508 and LS1034 cells were cultured in RPMI 1640 (GIBCO) for and HT55 Co and SK-CO1 cells in EMEM (Sigma) supplemented with 10% HI-FBS (heat-inactivated fetal bovine serum, GE Healthcare), 2 mM L-Glutamine 1% non-essential aminoacids (NEAA; GIBCO).CL-14 cells were cultured in DMEM/F12 (GIBCO) containing 20% HI-FBS, 2 mM L-Glutamine 1% NEAA, NCM460 in M3-base medium (INCELL) containing 10% HI-FBS,recombinant 293 LY6G6D^+^ in DMEM 10% HI-FBS, 2 mM L-Glutamine 1% NEAA and 1.0 mg/ml Geneticin (GIBCO) and Jurkat WT-NFATluc cells were cultured in RPMI 1640 (GIBCO), 10% HI-FBS, 2 mM L-Glutamine1% NEAA and 0.25 mg/ml Hygromycin (ThermoFisher Scientific).

293 cells (1x10^6^) were transfected with 5 µg DNA of LY6G6D_NFLAG_pcDNA 3.1(+) A009 (LifeTechnologies) by using Cell Line Nucleofector™ Kit V (Amaxa/Lonza) according to manufacturer’s instructions. After 24 hours incubation at 37°C, 5% CO_2_, 95% humidity, 1.0 mg/ml Geneticin was added to the medium. Cells were analyzed in FACS for LY6G6D expression.

Jurkat-E6.1 (from ATCC # TIB-152) were transfected with pGL4.30 NFATluciferase reporter plasmid (Promega cat. # E8481) according to manufacturer’s instructions

### Immunohistochemical analysis in human CRC

FFPE (formalin-fixed and paraffin embedded) tissue samples and TMA (Tissue Microarrays) were sliced into three micrometer sections and mounted on glass slides, stained with anti-LY6G6D clone 10C1 antibody or isotype control followed by OptiView DAB IHC Detection Kit (Roche Diagnostics). The stained tissue sections were evaluated by a pathologist including assessment of the percentage of stained tumor cells and the staining intensity. Sections containing at least 1% LY6G6D expressing tumor cells were scored as positive.

### Discovery, expression, purification and biochemical characterization of LY6G6D specific antibodies and LY6G6D

To identify monoclonal antibodies that specifically bind to the human LY6G6D protein, mice were immunized with a human-IgG1-Fc-His6 fusion protein derived from the matured human LY6G6D sequence (amino acid 20-103). Serum titers were measured by ELISA using an analogous His6 fusion protein. Splenocytes from responsive mice were pooled and immortalized by PEG based fusion. Positive clones were selected by ELISA and monoclonal hybridoma cells were generated by limited dilution. Binding was confirmed by ELISA from purified IgG obtained from hybridoma supernatant.

Next, two different screening processes were performed to select clones for IHC and TcE. To select clones for IHC and to ensure antibody specificity and selectivity in fixed tissues, clones were selected by staining of formalin-fixed paraffin-embedded cells mRNA positive/negative CRC cell lines. Clone 10C1 was selected eventually by this process. To select clones suitable as TcE and therefore to ensure recognition of LY6G6D in its native confirmation on live cells clones screened for binding to mRNA positive/negative CRC cell lines in FACS using unfixed cells. Clone 2C11A8 is derived from this later screen. Nucleotide sequence of 2C11A8 was determined after Vgene recovery.

The LY6G6D specific T cell engager was based on LY6G6D specific clone 2C11A8 and CD3ϵ specific clone SP34 sequences including a C-terminal hexahistidine (His6 tag). The DNA sequence was synthesized (Geneart) and cloned in the pTT5 expression vector (National Research Council, Canada). Trans IT PRO transfection reagent (Mirus Bio) was used for transient transfection of CHO-3E7 cells (National Research Council, Canada) and the TcE was expressed for 10 days. Clarified culture supernatant was purified by IMAC affinity chromatography (HisTrap FF, Cytivia). Purified TcE was formulated in 50 mM sodium acetate, 100 mM NaCl, pH 5.0, and analyzed by analytical size-exclusion chromatography (Agilent PL1580-5301 column) coupled to multi-angle light scattering, SDS-PAGE, and intact mass spectrometry. The LY6G6D specific TcE showed the expected molecular weight with >99% purity and <3% aggregated species.

Recombinant LY6G6D protein DNA was generated by gene synthesis (Geneart) and standard recombinant DNA technology. The protein was expressed in HEK293-6E cells by transient transfection using PEI MAX (Polysciences). Cells were harvested 96 hours post transfection, and supernatant was collected and clarified using G4 filter (Sartopore). The protein was purified from supernatant by IMAC chromatography (HisTrap FF, Cytivia) followed by size exclusion chromatography (Superdex 200 16/600, Cytivia). Purified protein was formulated in 1 x PBS, 0.2 M sucrose, 5% glycerol, 0.01% CHAPS, pH – 7.2 and quality and identity confirmed by analytical size-exclusion chromatography, SDS-PAGE, and intact mass spectrometry.

Affinity analysis of the LY6G6D specific TcE was performed by surface plasmon resonance using BIAcore T200 device (Cytiva). In brief, the TcE was immobilized on CM5 Chip (Cytiva) and recombinant LY6G6D protein was injected at concentrations of 0-9000 nM in HBS-EP (HEPES 10 mM,NaCl 150 mM, EDTA 3mM, 0.005% Tween-20). Measurements were run at 25°C and data were analyzed according to the 1:1 Langmuir model.

### PI-PLC treatment to cleave GPI anchor and remove LY6G6D from the cell membrane

Cells were harvested and incubated with 0.5-0.0001 units of PI-PLC (ThermoFisher) for 30 min at 37°C with slight shaking. Flow cytometry analysis were performed to confirm release of LY6G6D after PI-PLC treatment.

### Cytolysis assays

Target cells were seeded and incubated at 37°C. After 3 hours, purified T-cells from human peripheral blood mononuclear cells (EasySep™ Human T Cell Enrichment Kit, Stemcell) were added in a target to T cell ratio 1:10 with different concentrations of LY6G6D/CD3 Target cell killing was assessed after 48 or 72 hours by quantification of LDH released following manufacturer’s instructions (Cytotoxicity detection kit-Plus, Roche Applied Biosystems). Target cells with 3% Triton X-100 represents the maximum cell lysis (100%), and target cells with effectors cells the minimum lysis (0%). Percentage of specific cell lysis was calculated as [sample LDH release - spontaneous LDH release]/[maximum LDH release – spontaneous LDH release] x 100.

To determine the total number of LY6G6D positive and negative cells in the coculture experiments after treatment, each population was labelled with different CellTrace™ dyes (Invitrogen) according to manufacturer’s instructions. Cell lysis after 72 hours was determined by quantifying absolute live cells by flow cytometry.

To quantify the total number of cells after treatment, AccuCheck counting beads (Invitrogen) were added following manufacturer’s instruction before acquisition of samples on FACS Canto-II (BD Biosciences). Data was analyzed on FlowJo (TreeStar).

### Flow cytometry analysis

Expression of activation markers on T cells were analyzed at end-point of the cytolysis assays. T-cells were stained with Zombie Green Viability Dye or Zombie NIR Viability Dye (BioLegend) and surface antibodies against human CD4, CD8, CD69 and CD25 and CD107a (BD Biosciences or BioLegend). For expression of GranzymeB and perforin, cells were fixed and permeabilized with the Foxp3/Transcription Factor Staining Buffer Set (eBioscience/ThermoFisher) according to manufacturer’s instructions, and stained with anti- GranzymeB and anti-Perforin antibodies (eBioscience/ThermoFisher) for 30 min at 4°C.

To assess LY6G6D expression on target cells, they were incubated with Zombie NIR Fixable Viability Dye and anti-human LY6G6D-PE (**BioLegend)**

To determine the total number of viable LY6G6D positive or negative cells in the cocultures, total cells were stained with Zombie Viability Dye and anti-CD45 (BioLegend). Before acquisition, AccuCheck counting beads (Invitrogen) were added to the cell suspension. Cells were acquired on FACS Canto-II or Fortessa flow cytometer (BD Biosciences) and data analyzed on FlowJo (Tree Star).

### Quantification of LY6G6D density on cell lines

Quantification of LY6G6D cell surface molecules expressed on cell lines was performed by flow cytometry using the QIFIKIT^®^ (Agilent, Santa Clara, CA, USA) according of manufacturer’s instructions.

### Jurkat reporter assay

Target cells were seeded and 3 hours after LY6G6D/CD3 or untargeted control Fab-scFv,and reporter Jurkat NFATluc cells were added in a target to effector cell ratio of 1:10. After 24 hours of incubation at 37°C, activation of reporter cells was determined by luciferase expression (Steady-Glo^®^ Luciferase Assay System; Promega) on EnSight™ (PerkinElmer) accordingly to manufacturer’s instructions.

### Measurement of Caspase 3/7 activation

Cytolysis assay was performed as described above. LY6G6D negative and positive cells labelled with cell tracer FarRed or cell tracer violet dye (BioLegend), respectively. T cells and Caspase 3/7 green dye (Sartorius) were added and cultured with or without TcE. Images of cells were acquired on an Opera Phenix Plus device (PerkinElmer) every 3 hours for 24 hours. Analysis of the images were performed using the Harmony Software, and display of images were done by the TIBCO Spotfire Software 4.9.

### CRC Precision Cut Tumor Slice Cultures

Freshly excised tumor tissue was transferred to the lab within 30 min after surgery and 200 μm sections were prepared using a Compresstome^®^ VF-300-0Z. Tumor slides were incubated with LY6G6D/CD3 TcE or TNP/CD3 control at 37°C, 5% CO2 for 48 hours. Then fixed in formalin and embedded in paraffin. LY6G6D and CD3 expression were analyzed by immunohistochemistry in baseline slices; Granzyme B was analyzed from treated tumor slices. IFN-γ, Granzyme B, IP-10, IL-2, TNFα and MCP-1 were quantified in the cell supernatant using U-PLEX Assays (Mesoscale Discovery, Rockville, MA, USA) following the manufacturer’s instructions. All slice culture experiments were performed in biological duplicates or triplicates. Cytokines were quantified from all biological replicates, the mean value of each sample was then normalized to the respective untreated (medium only) samples and depicted as fold change.

### Tumor model and monitoring of tumor growth

All mice were housed and treated according to approved IACUC protocols. NOG mice were injected s.c. with 1 x 10^6^ LS1034 cells. When tumor size reached 100 mm3, mice were injected i.v. with 1 x 10^6^ human PBMC. Treatment with TcE started 3 days after PBMC transfer, 0.5 mg/kg of LY6G6D/CD3 Fab-scFv negative control TNP-CD3 Fab-scFv were administered daily for 5 days per week. Animals were monitored daily for morbidity and mortality. Tumor volumes were measured twice per week with digital caliper, and the volume will be expressed in mm^3^ using the formula: “V = (L x W x W)/2, where L is tumor length and W is tumor width.

### Organotypic cultures

Isolated or cocultured NCM460D and LS1034 cells were seeded at 1000 cells/well in ultra-low attachment 96 well plates (costar) using RPMI (Gibco) supplemented with 10% FBS (HyClone), and incubated for three days at 37°C, 5% CO_2_.

After confirming spheroid formation by microscope 5000 PBMCs/well (StemCell Technologies) and treatments were added. Treatments consisted of LY6G6D/CD3 TcE at different concentrations. For blocking experiments, 10µg/ml Entanercept (EDQM), 5µg/ml CD178 monoclonal antibody (Invitrogen), or a combination or 1µg/ml human IFNγ antibody (bio-techne) with 1µg/ml human IFNγR antibody (bio-techne) were added. PBMCs and compounds were diluted with RPMI supplemented with 10% FBS. Spheroid growth was measured with an IncuCyte S3 (Sartorius) for the next 7 days and data analyzed bythe IncuCyte2021C software.

### Statistical analysis

Statistical analysis was performed using GraphPad Prism 9.0.0 software. A one-sided non-parametric Mann-Whitney- U-test was applied to compare treated animals with LY6G6D/CD3 TcE ITE and TNP/CD3 TcE control. ** p < 0.01.

## Results

### Identification of LY6G6D as a CRC selectively expressed antigen

Potent re-direction of cytotoxic T-cells to tumors requires tumor selectively expressed antigens. Using public transcriptomic data, we identified LY6G6D as a CRC specific antigen within 11616 tumor samples from the Cancer Genome Atlas (TCGA), and 17588 normal samples from TCGA (normal adjacent, n=2150) and the Genotype-Tissue Expression project (GTEx, n=15438) (Supplemental Fig. 1A). This data confirms the recent identification of LY6G6D as a CRC marker, mainly expressed in MSS tumors (Supplemental Fig. 1B), by a similar in silico approach (7).

**Figure 1:**
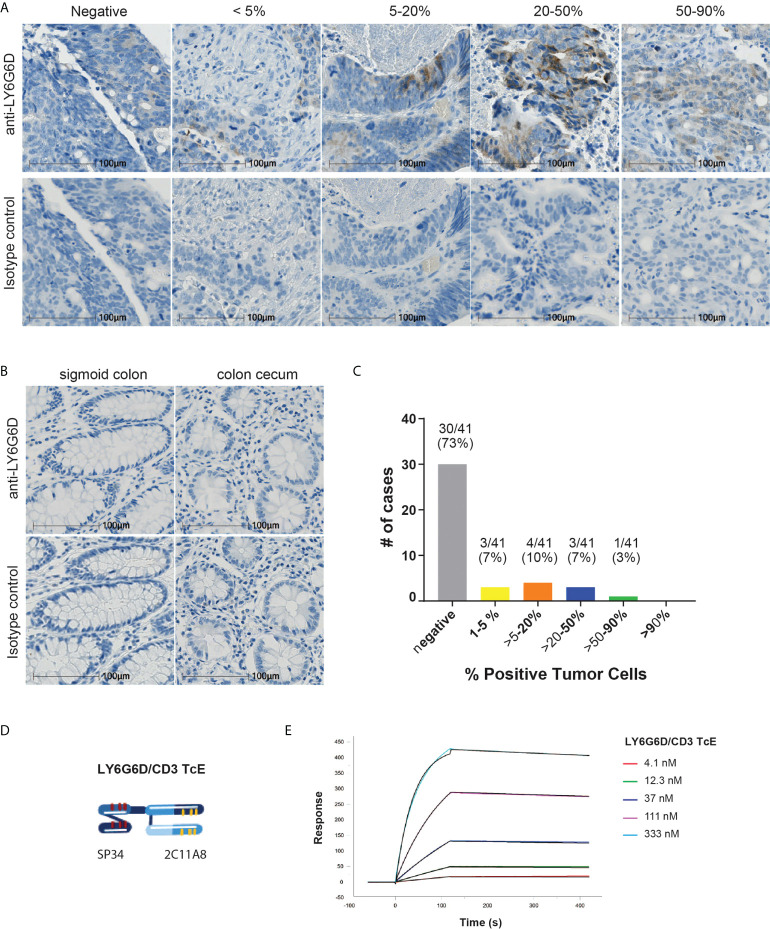
LY6G6D is a tumor antigen specifically expressed in CRC. **(A-C)** LY6G6D IHC staining. Representative images of CRC samples **(A)** and normal colon tissue **(B)**. Graph depicting the percentage of positive cells in CRC samples (n=41). **(D)** Schematic graphic of LY6G6D/CD3 TcE. The Fab-scFv TcE format contains a N-terminal 2C11A8 derived Fab linked with *via* (G4S)4 linker to a C-terminal CD3ϵ specific scFv derived from the murine clone SP34. **(E)** Surface plasmon resonance analysis of LY6G6D specific TcE. TcE was immobilized on CM5 chip, recombinant LY6G6D protein was used as analyte, and data were analyzed by a 1:1 Langmuir model. KD=2.5 nM is for LY6G6D, ka=6.9x104 1/M*s and kd=1.7x10-4 1/s.

To demonstrate the specific expression of LY6G6D in tumors we developed an immunohistochemistry assay to study its distribution. Specific antibodies were generated by immunizing mice with recombinant protein derived from the matured human LY6G6D protein sequence. Clone 10C1 was selected to study the prevalence of LY6G6D in colorectal cancer (CRC), as its staining was blocked with 50x recombinant LY6G6D protein, confirming its specificity for LY6G6D (Supplemental Fig. 2A-B). The abundance of LY6G6D expression was analyzed in tumor samples of a CRC TMA (n=41) and non-malignant colon tissue using clone 10C1. We observed that similarly to other tumor specific antigens, LY6G6D expression was heterogeneous within the tumor area, and the percentage of positive cells ranged from 1 to 90% of the tumor content (**Figure 1A** and **Supplemental 2C**), while LY6G6D was not detected in normal colorectal tissues (**Figure 1B**). Eleven samples out of 41 showed a positive staining, indicating a prevalence of LY6G6D expression of 27% in this cancer sample cohort (**Figure 1C**). Overall, we concluded that LY6G6D is expressed in a significant percentage of CRC samples and could be a good target for T-cell engager, with the aim of redirecting T cells into CRC tumors, induce T cell activation and subsequent tumor cell killing.

### Generation and characterization of LY6G6D specific T-cell engager

The LY6G6D specific murine IgG clone 2C11A8 was selected and reformatted into a bispecific T-cell engager format Fab-scFv which has been described previously (14). Here, the Fab-scFv (**Figure 1D**) consists of the N-terminal 2C11A8 derived Fab linked *via* the (G4S)4 linker to a C-terminal CD3ϵ specific scFv derived from the murine clone SP34 (15). The Fab-scFv T-cell engager was expressed in Chinese hamster ovary cells, purified, and its purity and integrity was confirmed by SDS-PAGE, analytical size exclusion chromatography, and mass spectrometry. The Fab-scFv T-cell engager has an affinity of KD=2.5 nM for LY6G6D as determined by SPR (**Figure 1E**).

### T cell activation and cytotoxicity correlates with level of LY6G6D in the cell membrane

**Figure 2:**
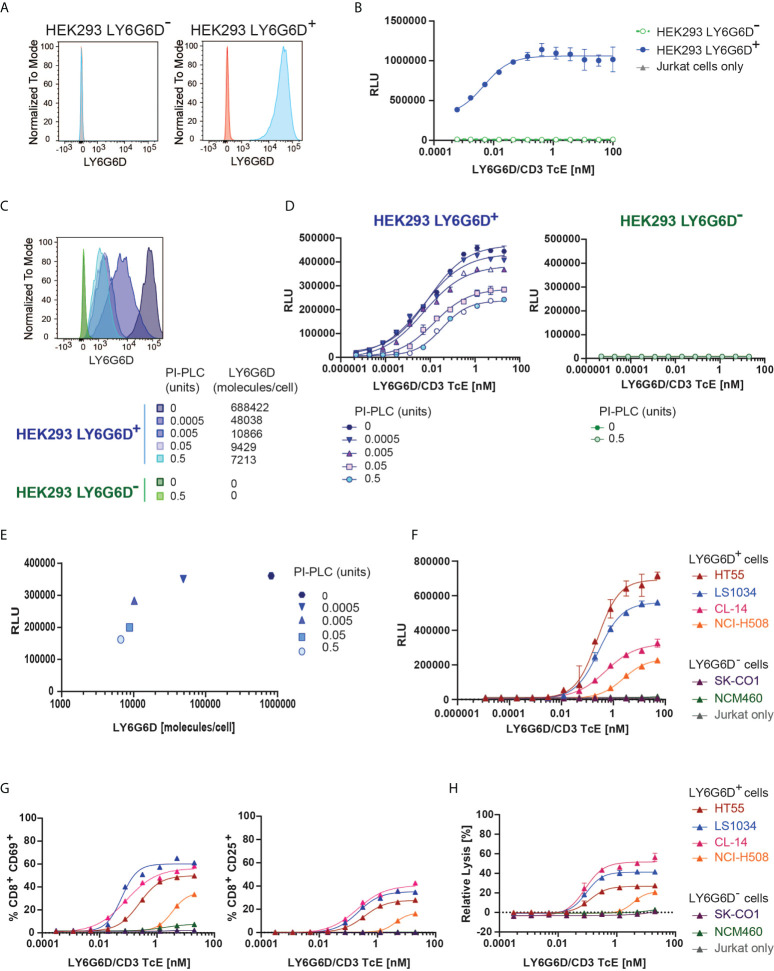
*In vitro* efficacy and specificity of LY6G6D/CD3 TcE. **(A)** LY6G6D expression in parental and LY6G6D transfected HEK293 cells **(B)** Jurkat cells and HEK293 LY6G6D^-^ or HEK293 LY6G6D^+^ cells were co-cultivated in the presence of increasing concentrations of LY6G6D/CD3 TcE for 24 hours. **(C)** LY6G6D density in HEK293 LY6G6D^+^ and HEK293 LY6G6D^-^ cells pre-incubated with PI-PLC for 30 min. **(D)** PI-PLC pre-incubated HEK293 cells and Jurkat cells were co-cultivated in presence of increasing concentrations of LY6G6D/CD3 TcE for 24 hours. **(E)** Correlation between LY6G6D density and activation of Jurkat cells. **(F)** Activation of Jurkat cells after 48 hours of incubation with LY6G6D-positive (HT55, LS1034, CL-14 and NCI-H508) or negative (SK-CO1 and NCM460) tumor cells and increasing concentrations of LY6G6D/CD3 TcE. **(G, H)** LY6G6D-positive (HT55, LS1034, CL-14 and NCI-H508) or negative (SK-CO1 and NCM460) tumor cells were co-incubated with purified T cells and increasing amounts of concentrations of LY6G6D/CD3 TcE. Killing of tumor cells **(G)** and activation of T cells **(H)** were assessed after 72 hours of incubation.

We first tested the specificity of the LY6G6D/CD3 TcE using Jurkat WT-NFAT luc reporter cells, as a readout for T cell activation upon cross-linking of CD3. Activation of Jurkat cells triggered by the LY6G6D/CD3 TcE was only observed in the presence of LY6G6D^-^transfected HEK293 (HEK293 LY6G6D^+^), but not when the parental HEK293 (HEK293 LY6G6D^-^) cells were used (**Figures 2A, B**).

LY6G6D is anchored covalently to the cell membrane by a glycosylphosphatidylinositol (GPI) molecule. The GPI-anchored proteins (GPI-AP) can be released from the cell membrane by phospholipases and putative substrate-specific GPI-AP-releasing enzymes (16). We mimic a potential release of the target in the tumor site by incubating the cells with a wide concentration range from 0.0005 to 0.5 units of phosphatidylinositol (Pl)-specific phospholipase C (PI-PLC) from Bacillus thuringiensis. The LY6G6D density on the cell surface of HEK293 LY6G6D^+^ cells was gradually reduced, but even in the highest PI-PLC concentration, LY6G6D was not completely removed (**Figure 2C**). The LY6G6D/CD3 TcE potency, measured by Jurkat WT-NFAT luc reporter cell activation, correlated with LY6G6D density (**Figures 2D, E**). We then selected LY6G6D positive and negative CRC cell lines (Supplemental Fig. 2A) to confirm the activation of Jurkat cells in the context of endogenous LY6G6D expression. CRC cell lines were cocultured with Jurkat WT-NFAT luc reporter cells in a wide concentration range of the LY6G6D/CD3 TcE. Endogenous LY6G6D expression on HT55, LS1034, CL-14 and NCI-H508 tumor cells was sufficient to activate Jurkat WT-NFAT luc reporter cells, while no activation was observed in the presence of the LY6G6D negative CRC cell lines SK-CO1 and NCM-460, or in the absence of tumor cells, confirming the selectivity of the LY6G6D/CD3 TcE (**Figure 2F**). Finally, we also demonstrated the efficacy of the LY6G6D/CD3 TcE in CRC cell lines co-incubated with purified human T cells from PBMCs. Activation of CD8 T cells measured by expression of CD25 and CD69, T-cell proliferation and expression of the degranulation marker CD107, Perforin and Granzyme B was observed only in the presence of LY6G6D positive cell lines (**Figure 2G** and **Supplemental Figure 2B, C**). T-cell mediated killing was only observed on LY6G6D positive cell lines, with a EC_50_ ranging from 0.1 to 1 nM (**Figure 2H**).

### Activity of LY6G6D/CD3 TcE in ex vivo patient-derived CRC tumor slice cultures

**Figure 3:**
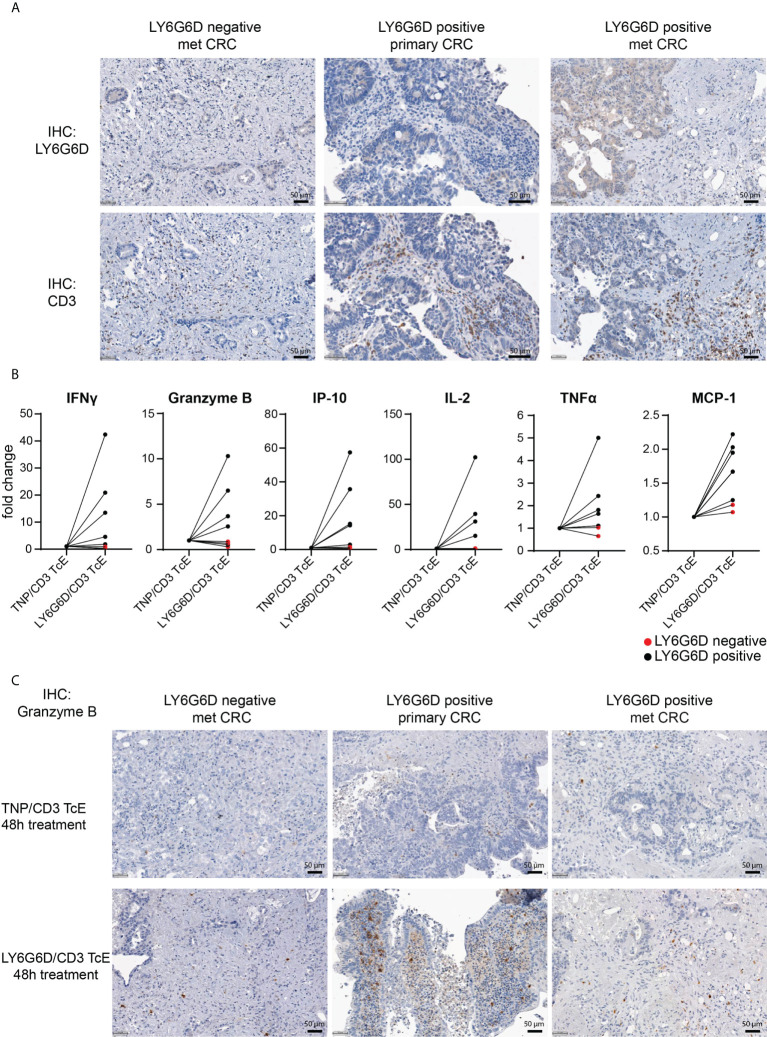
*Ex vivo* activation of T cells in LY6G6D positive CRC Precision Cut Tumor Slice Cultures. **(A)** Baseline expression of LY6G6D and CD3 expression in representative samples. **(B)** Fresh tumor tissue slides from eight CRC patients were incubated for 48 hours with 1 nM LY6G6D/CD3 TcE or control TNP/CD3 TcE. After 48 hours IFN-γ, Granzyme B, IP-10, Il-2, TNFα and MCP-1 were quantified in the cell culture supernatant. Each dot shows one CRC patient sample whereas red dots indicate LY6G6D negative tumor samples. **(C)** Representative images of Granzyme B staining in tumor slides after 48 hours of incubation with 1 nM LY6G6D/CD3 TcE or control TNP/CD3.

We interrogated whether the LY6G6D/CD3 TcE could activate tumor infiltrating lymphocytes (TILs) in precision cut tumor samples from CRC patients. For this, eight CRC tissue slices were selected: five with LY6G6D expression and three LY6G6D negative (**Supplemental Figure 4A**). Basal expression of LY6G6D and infiltration of T cells was assessed by immunohistochemistry in fresh tumor tissue slices fixed in formalin (**Figure 3A**).

Tumor slices were incubated with 1 nM LY6G6D/CD3 TcE or TNP/CD3 TcE, a control TcE that binds to the irrelevant antigen trinitrophenol, for 48 hours and activation of TILs was assessed by quantifying cytokines in the cell culture supernatant and by staining the tumor slices with Granzyme B. Treatment with LY6G6D TcE induced more than 1.5-fold secretion of IFN-γ, IP-10 (5/5 LY6G6D positive samples), and Granzyme B, IL-2, TNFα and MCP-1 (4/5 LY6G6D positive samples) (**Figure 3B**). Immunohistochemistry of Granzyme B after the incubation was also observed in LY6G6D positive samples (**Figure 3C**). To demonstrate that the lack of activation of TILs in LY6G6D negative tumors is due to the absence of target expression, we incubated one of the CRC samples with an EpCAM/CD3 TcE as all the selected tumors expressed EpCAM. In this case the EpCAM/CD3 molecule induced Granzyme B on TILs and the secretion of cytokines in the culture media (Supplemental Fig. 3 A-C), demonstrating that the LY6G6D/CD3 TcE is specific, and the lack of T cell activation is not due to a defect on T cells, since they can be activated in the presence of EpCAM/CD3 TcE in EpCAM positive tumor samples.

### LY6G6D/CD3 TcE induced anti-tumor activity and changed the TME

**Figure 4:**
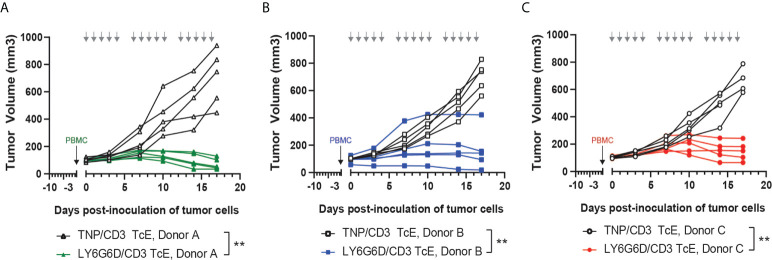
*In vivo* efficacy of LY6G6D/CD3 TcE. **(A-C)** Anti-tumor activity of LY6G6D/CD3 TcE in established LS1034 xenograft tumors in NSG mice. Mice were humanized with human PBMCs at randomization, and treated with 0.5 mg/kg LY6G6D/CD3 TcE or control TNP/CD3 five days per week. Each graph shows data from one out of three independent PBMC donors. Days of treatment are annotated by gray arrows. **p<0.01.

To test the *in vivo* efficacy of the LY6G6D/CD3 TcE, PBMC-humanized NSG mice bearing human LY6G6D positive LS-1034 xenografts were treated with LY6G6D/CD3 TcE or TNP/CD3 TcE. LY6G6D/CD3 TcE does not contain an Fc domain and lacks an extended half-life therefore animals were dosed daily, five times a week. Treatment started three days after the inoculation of human PBMCs when the mean tumor volume was 100 mm^3^. Treatment with LY6G6D/CD3 TcE led to 90% tumor growth inhibition (p < 0.01) and tumor regression was observed in 2 out of 5 animals reconstituted with PBMCs from donor A (**Figure 4A**). Two additional donors were used to humanize the NSG mice to determine the influence of different PBMC donors on tumor control. Similar tumor control was observed with donor B and C, (p < 0.01) demonstrating that the LY6G6D/CD3 TcE induces efficient tumor control in LY6G6D positive tumors (**Figure 4B, C**).

### LY6G6D/CD3 TcE induced bystander cell killing of target-negative cells

**Figure 5:**
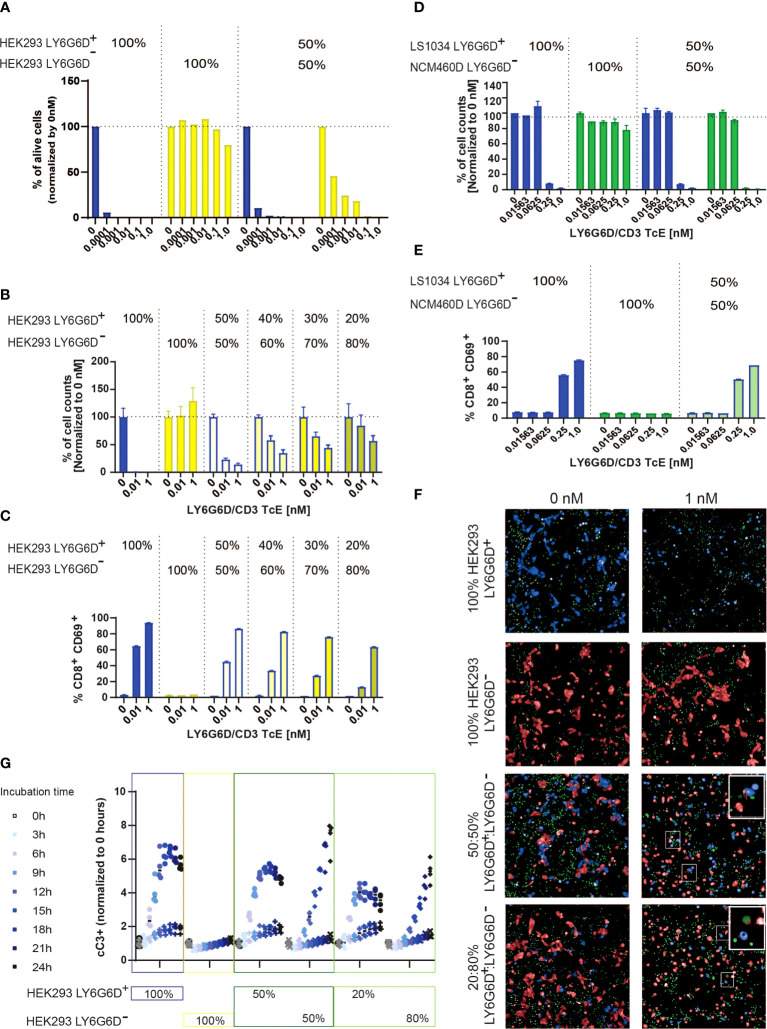
LY6G6D/CD3 TcE induces bystander killing of LY6G6D^-^ cells *in vitro*. **(A, B)** HEK293 LY6G6D^+^ and LY6G6D^-^ were labeled with cell tracer, plate isolated (100%) or cocultured at 1:1 ratio **(A)** or at different ratios **(B)**. Isolated or cocultured HEK293 cells were then co-incubated with purified T cells and increasing amounts of concentrations of LY6G6D/CD3 TcE. After 72 hours, the total number of HEK293 LY6G6D^+^ and LY6G6D^-^ were counted and the percentage of viable cells were calculated by normalizing with non-treated cells in each condition. **(C)** Activation of T cells after 48 hours of co-incubation with HEK293 LY6G6D^+^ and LY6G6D- cells isolated or cocultured under different ratios. **(D, E)** LS1034 and NCM460D tumor cells were labeled with cell tracer, plate isolated (100%) or cocultured at 1:1 ratio. Tumor cells were then co-incubated with purified T cells and increasing amounts of concentrations of LY6G6D/CD3 TcE. The total number of tumor cells **(D)** and T cell activation **(E)** was analyzed after 48 hours of incubation. **(F, G)** HEK293 LY6G6D^+^ (blue) and LY6G6D^-^ (red) were labeled, plate isolated (100%) or cocultured and co-incubated with purified T cells, increasing amounts of LY6G6D/CD3 TcE and caspase 3/7 green dye. Confocal microscopy images of cultures were acquired over time **(F)**, and apoptotic or double stained HEK293 LY6G6D^+^ (blue/green) and LY6G6D^-^ (red/green) cells were quantified **(G)**.

LY6G6D expression in CRC showed considerable levels of spatial heterogeneity as shown in **Figures 1C** and **3A**. The heterogeneous target expression is observed for other tumor specific antigens (17) and poses an obstacle for the therapeutic effect of a specific TcE. To recapitulate the heterogeneous expression of the target *in vitro* we cocultured HEK293 LY6G6D positive and negative cells and tested the killing effect of the LY6G6D/CD3 TcE in both subpopulations. As expected for monocultures, LY6G6D-positive cells were killed in the present of the TcE and T cells, while the LY6G6D-negative cells were not affected; however, when both cell types where cocultured in a 1:1 ratio the LY6G6D-negative cells were killed in a dose dependent manner (**Figure 5A**). We investigated whether the bystander effect on negative target cells was dependent on the percentage of positive target cells in the coculture. For this purpose, we kept the same total number of cells but lowered the percentage of LY6G6D-positive cells from 50% to 20%. The level of killing of LY6G6D-negative cells decreased in parallel with the reduction of target-positive cells in the coculture (**Figure 5B**). Activation of T cells is also decreased upon reduction of target positive cells in the coculture. Nevertheless 20% of positive cells in the coculture is enough to activate T cells (**Figure 5C**). The bystander effect was also studied in cells with endogenous expression of LY6G6D. For this purpose, we cocultured LS1034 and NCM460D cells, as target positive and negative cells, respectively. Both, killing of target negative NCM460D cells and T cell activation were only observed in the presence of target positive LS1034 cells (**Figures 5D, E**). The bystander effect is not a unique effect of this specific target and TcE, since EpCAM/CD3 TcE also induced killing of EpCAM-negative cells in coculture with EpCAM-positive cells (**Supplemental Figures 5A, B**).

To understand the kinetics of cell death of the two cell subpopulations in the coculture, LY6G6D positive and negative cells were cell-trace labelled and caspase 3 activation was monitored every three hours by confocal microscopy. As expected, cleaved caspase 3 was scarcely found in the isolated HEK293 LY6G6D^-^ cells but was clearly shown in the coculture with HEK293 LY6G6D^+^ cells (**Figure 5F**). Quantification of caspase 3 activation overtime showed some differences in the kinetics of cell killing negative targets cells needed at least 9 hours to get the same level of caspase 3/7 positive cells (**Figure 5G**). These data imply that killing of negative target cells occurs after T cells are activated upon TcE engagement.

### Bystander effect is observed in organotypic cultures and it is cytokine-dependent

**Figure 6:**
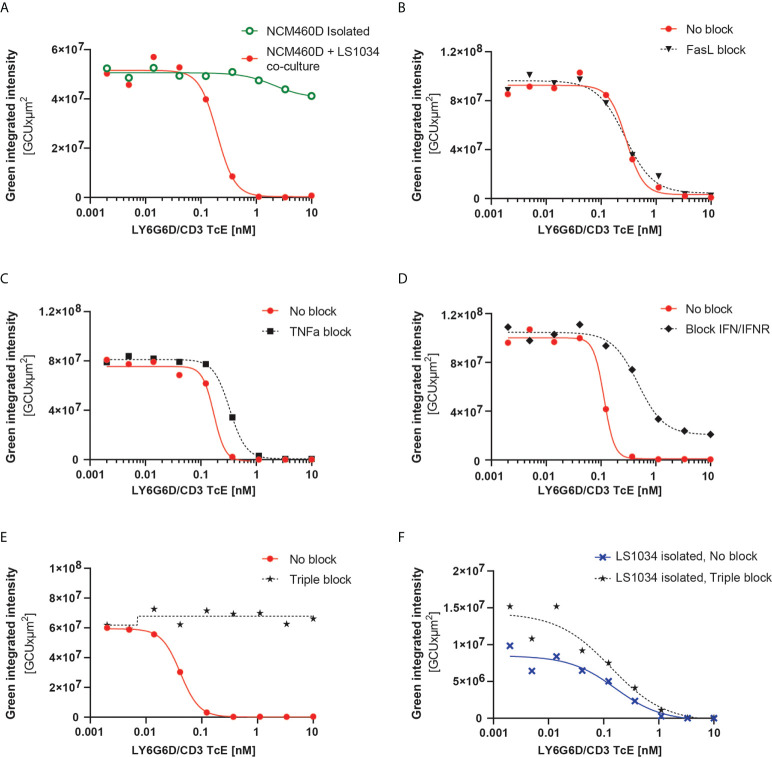
LY6G6D/CD3 TcE induces bystander killing of LY6G6D^-^ cells in 3D spheroids. **(A)** Image analysis of spheroids containing NCM460D cells or a 1:1 coculture of NCM460D and LS1034 cells. Spheroids were incubated with PBMCs (1:5 ratio, T:E) and increasing amounts of LY6G6D/CD3 TcE for 7 days. Total green signal at day 7 from NCM460D cells is shown. **(B-E)** Spheroids containing a 1:1 coculture of NCM460D and LS1034 cells, PBMCs (1:5 ratio, T:E), increasing amounts LY6G6D/CD3 TcE and 10 µg/ml CD178 monoclonal antibody (Invitrogen) **(B)**, 10µg/ml Entanercept **(C)**, 1µg/ml human IFNγ antibody with 1µg/ml human IFNGR antibody **(D)** or a combination with all blocking compounds **(E)**. Total green signal at day 7 from NCM460D cells is shown. **(F)** Image analysis of spheroids containing LS1034 cells. Spheroids were incubated with PBMCs (1:5 ratio, T:E), increasing amounts of LY6G6D/CD3 TcE for 7 days or a combination with all blocking compounds like in Figure **(E)** Total green signal at day 7 from LS1034 cells is shown.

We further tested the bystander effect in a 3D scenario, where the arrangement of cells is more representative of real tumors. For this purpose, organotypic cultures of LS1034 and NCM460D-GFP cells in a 1:1 ratio or NCM460D-GFP alone were generated, and the killing of LY6G6D-negative cells was monitored over time. As observed in the 2D culture, NCM460D-GFP cells were killed in the coculture but not in the monoculture (**Figure 6A** and **Video 01**).

The mechanism overlying the bystander effect in TcE and CAR T cell therapeutics has been studied recently. Fas/FasL, TNFα and IFNγ signaling have been described as possible mechanisms, albeit none (18–20) sufficient to explain the bystander effect in all circumstances or models [18–20]. To understand the mechanism responsible for the bystander killing in our system, we blocked pharmacologically and individually the three pathways in our tumor cell 3D cocultures containing LS1034 and NCM460D-GFP cells. Even though NCM460D cells express FAS in basal conditions (Supplemental Fig. 6A), rescue of the killing of NCM460D-GFP cells in the coculture was not achieved by blocking this pathway (**Figure 6B**). Both TNFα and IFNγ are secreted by T cells in presence of the TcE and LY6G6D positive cells (Supplemental Fig. 6B). Blocking these two pathways led to different outcomes: while TNFα blockade had a little effect (**Figure 6C**), interfering with the IFNγ pathway partially prevented the killing of NCM460D-GFP cells (**Figure 6D**). Furthermore, simultaneous blockade of these three pathways completely abolished the bystander killing of target negative cells, even at the highest concentration of the TcE (**Figure 6E**). In order to elucidate whether this impaired bystander killing was due to interference with the target-specific effects, we used the blocking agents in a monoculture of target positive LS1034 cells. We observed that the killing of LY6G6D^+^ triggered directly by the TcE is independent on FAS, TNFα and IFNγ (**Figure 6F** and **Video 02**). This data demonstrates that the target-mediated tumor cell killing and the death of bystander target negative cells is mediated by different mechanisms.

## Discussion

One of the principal challenges in the development of TcE for solid tumors is the identification of truly tumor specific antigens that are not expressed in vital normal tissues. In contrast to solid tumors, hematological malignancies present lineage antigens like CD19, that can be targeted without the life-threatening consequences, as non-malignant cells expressing these antigens are dispensable (e.g. B-lymphocytes). However, these antigens are scarce in solid tumors, and the field is searching for antigens selectively expressed in tumor cells, to guarantee a thesrapeutic window (21). Therefore, there is a need for the identification of highly specific tumor antigens to increase the therapeutic window of TcE that have a potent mechanism of action. However, a highly selective target is usually expressed very heterogeneously among patients and within the same tumor (22).

In this study, we describe the identification of LY6G6D as a high tumor specific antigen in CRC, specifically in MSS tumors, confirming the same observations made by other groups (7, 23). This is an important finding since MSS is the most abundant CRC subtype and presents a poor response rate to immuno-oncology treatments (24). As confirmed by immunohistochemistry analysis by Wang et al. in a recent publication (23), the expression distribution of LY6G6D makes this target a rare tumor specific antigen with differential expression in CRC versus normal tissues, which allows the generation of a TcE with very restricted on-target/off-tumor toxicity. To further demonstrate the relevance of LY6G6D, we performed a prevalence analysis in CRC samples by immunohistochemistry and confirmed that 27% of the samples express LY6G6D on tumor cells.

To evaluate the suitability of LY6G6D as a target for redirecting T cells into tumors we generated a TcE and performed *in vitro* and *in vivo* assays to validate its specificity and efficacy. In a homogeneous population of LY6G6D positive cells, the LY6G6D/CD3 TcE induced potent tumor cell lysis and T cell activation which correlated with target density in the cell membrane. *In vivo* studies showed strong tumor regressions in all animals treated with LY6G6D/CD3 TcE monotherapy. Tumors from treated animals presented a more inflamed phenotype, with higher infiltration of T cells (data not shown), as observed by others and our group previously (11). This data demonstrated the efficacy and specificity of theLY6G6D/CD3 TcE in commonly used cell-based assays and *in vivo* models to characterize TcE molecules. However, these systems do not interrogate the impact of the tumor microenvironment and the native architecture of tumors in the activity of drugs. For this purpose, patient-derived *ex vivo* models are more physiologically relevant approaches (25). In Precision Cut CRC Tumor Slice Cultures, treatment with LY6G6D/CD3 induced activation of tumor infiltrating T cells, measured by released inflammatory cytokines and granzyme B, only in LY6G6D-positive tumors. Target cell killing induced by TcE resembles the mechanism triggered by TCR-peptide-MHC interaction, leading to the formation of a lytic synapse (26). Indeed, granzyme B and perforin were induced by the LY6G6D/CD3 TcE only when T cells were cocultured with LY6G6D-positive tumor cells. Although this is the main mechanism mediating tumor control by TcE, some preclinical studies have reported the evidence of a TcE-bystander killing of target negative cells (20). Cytokine release, mainly IFNγ and TNFα (19, 27, 28), but also the Fas/FasL pathway, have been described to be the main mechanisms involved in bystander killing (18, 20). In 2D cell cocultures, LY6G6D/CD3 TcE induced activation of T cells and killing of LY6G6D-negative cells even at a low percentage of 20% LY6G6D-positive cells. Parallel overtime monitoring of activated caspase 3/7 demonstrated that killing of LY6G6D-negative cells is only delayed by nine hours approximately. To understand the mechanism in a more complex set-up, blockade assays in 3D organotypic assays demonstrated that none of the previously identified pathways are totally sufficient to mediate the bystander effect, since killing of LY6G6D negative cells was only completely prevented with concomitant blockade of Fas/FasL, TNα and IFNγ/IFNGR pathways. However, we corroborate that the lytic synapse should govern the direct killing of LY6G6D-positive tumor cells, since these cells were not protected from killing by the triple blockade. Demonstration of the bystander effect driven by another TcE has been also demonstrated by others *in vivo* using a EGFR TcE (20). In this study coimplanted luciferase-labeled EGFR^-^ tumor cells decreased within the tumors of EGFR TcE treated animals but not in animals treated with an irrelevant TcE. Therefore, effectively therapeutic potentiation of bystander killing may be an approach to counteract the heterogeneous expression and downregulation of tumor specific antigens to maximize the efficacy of T-cell redirecting therapies.

## Data availability statement

The datasets presented in this study can be found in online repositories. The names of the repository/repositories and accession number(s) can be found below: https://www.ncbi.nlm.nih.gov/gap/, phs000178.v11.p8 https://www.ncbi.nlm.nih.gov/gap/, phs000424.v8.p2.

## Ethics statement

The animal study was reviewed and approved by The protocol and any amendment(s) or procedures involving the care and use of animals for *in vivo* studies were reviewed and approved by the Institutional Animal Care and Use Committee (IACUC) of CrownBio prior to execution. During the study, the care and use of animals were conducted in accordance with the regulations of the Association for Assessment and Accreditation of Laboratory Animal Care (AAALAC). Short-time culture of human tumor samples was reviewed and approved by the Ethics Committee of the Medical University of Vienna (EK:2042/2019). Informed written consent was obtained from all participants in this study.

## Author contributions

LC: Designed the manuscript, supervised the study, and wrote the manuscript. SH: supervised antibody campaigns and prevalence studies. KM: Executed and analyzed *in vitro* experiments and contributed to the preparation of the figures. NS: Designed the molecule and established the production process. IT: Supervised and coordinated organotypic *in vitro* experiments. K. Fuchs: Designed and supervised the antibody generation campaigns. BT: Executed and analyzed organotypic *in vitro* experiments. CW: Screened specific antibodies. KB: Screened specific antibodies. MF: Planned, executed, and interpreted tissue slice culture experiments. MB: Planned tissue slice culture experiments. CB: Excised tumor samples and supported pathological examinations of slice cultures. PC: Supported histological analysis of CRC samples in ex vivo studies. AV: Supervised and coordinated ex vivo studies. PA: Supported the design, supervised, and evaluated the manuscript. All authors contributed to the article and approved the submitted version.

## Funding

This study was supported by Boehringer Ingelheim and the Basisprogramm grants of the Austrian Research Promotion Agency (FFG; 860968, 869530, 875923, and 883369).

## Acknowledgments

We thank Margit Zehetner, Andreas Schrenk, John Miglietta, Carolina Klicka, Sam Lukowski, Abdallah Souabni, Lisa-Sophie Heinz, Rene Hude and Katharina Kauer for their experimental support and help for this study; and our collaborator from Precision Antibody (A&G Pharmaceutical, Inc., Columbia, MD, USA) for Ab generation of 10C1 and from Syngene (International Ltd, Bangalore, India) for generation of 2C11A8.

## Conflict of interest

LC, SH, KM, NS, IT, KF, BT, CW, KB, PC, AV, and PA are employees of Boehringer Ingelheim affiliates. MB received research funding and speakers fee by Boehringer Ingelheim and Bristol Myers Squibb.

The remaining authors declare that the research was conducted in the absence of any commercial or financial relationships that could be construed as a potential conflict of interest.

## Publisher’s note

All claims expressed in this article are solely those of the authors and do not necessarily represent those of their affiliated organizations, or those of the publisher, the editors and the reviewers. Any product that may be evaluated in this article, or claim that may be made by its manufacturer, is not guaranteed or endorsed by the publisher.
