# Supplementary material for: LY6G6D is a selectively expressed colorectal cancer antigen that can be used for targeting a therapeutic T-cell response by a T-cell engager
Source: Front Immunol. 2022 Sep 8;13:1008764. doi: 10.3389/fimmu.2022.1008764 (PMC9493073; doi:10.3389/fimmu.2022.1008764)
Supplement: Supplementary file 1 [file Presentation_1.pdf]

Supplemental Figure and Material and Methods

Supplemental Figure 1

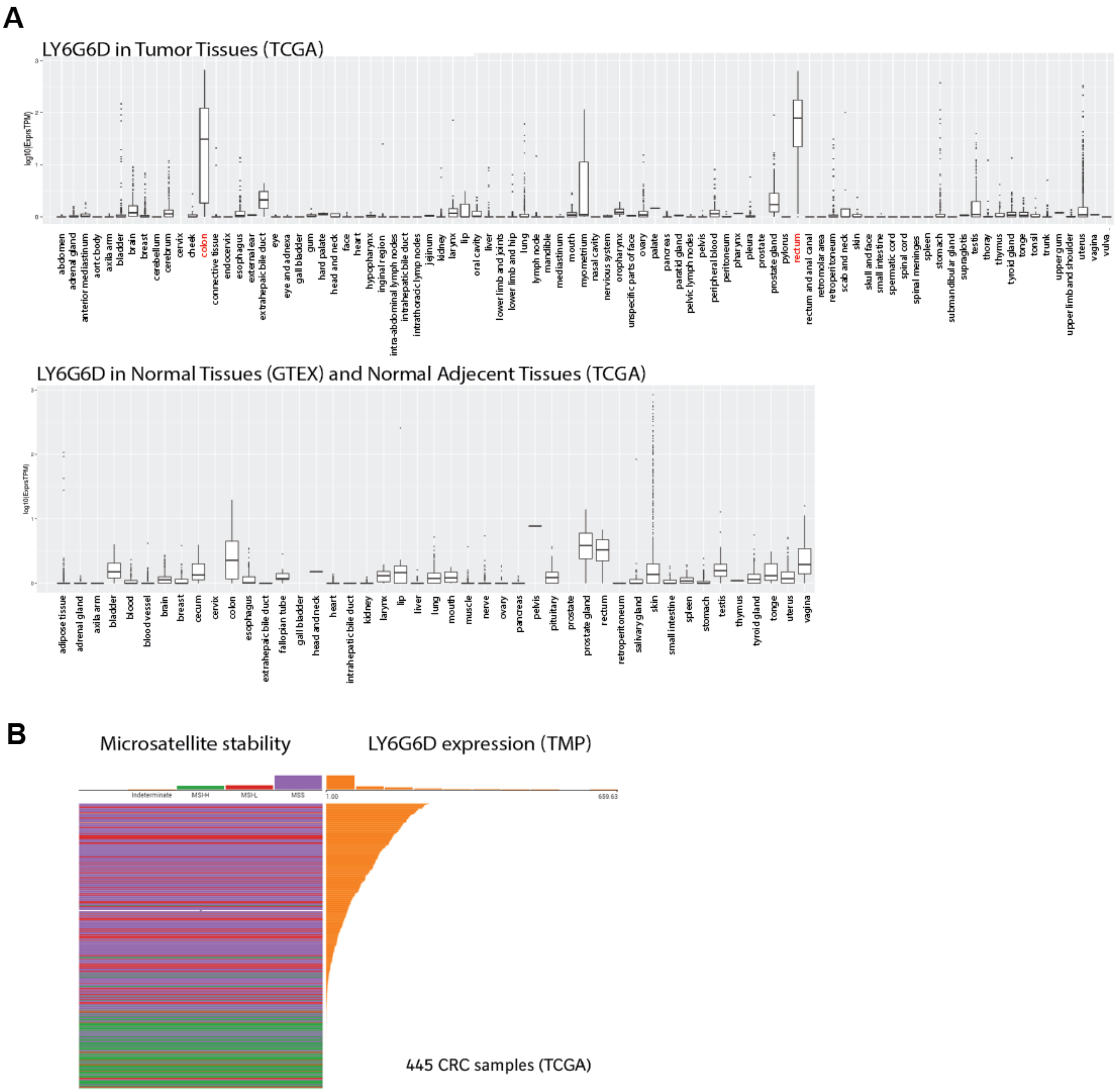

Supplemental Figure 1.

(A) LY6G6D mRNA expression in tumors (TCGA) and normal tissues (GTEX). (B) Correlation of LY6G6D mRNA expression and microsatellite instability status. MSI-H: microsatellite instability high (green); MSI-L: microsatellite instability low (red); MSS: microsatellite stable (purple).

Supplemental Figure 2

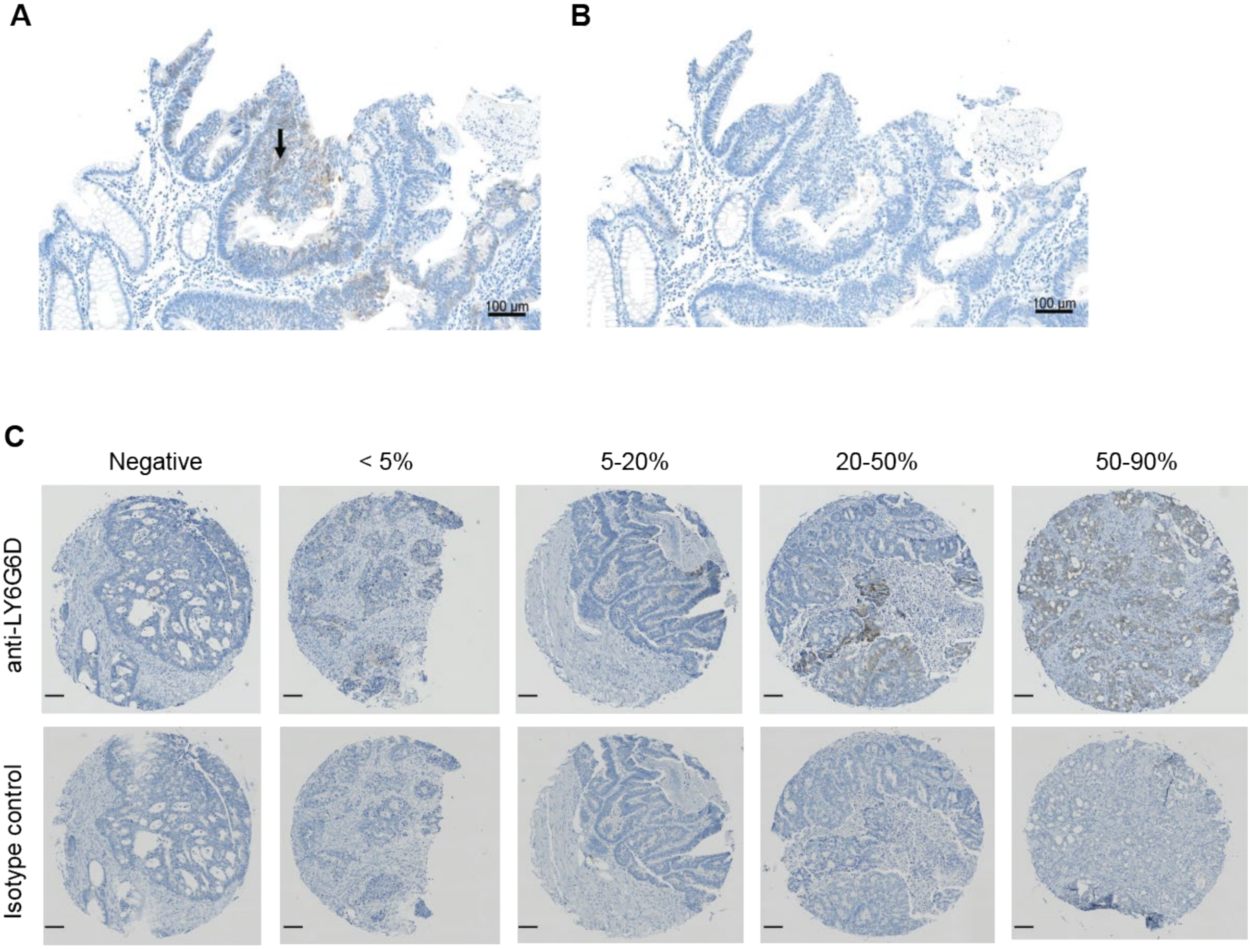

Supplemental Figure 2.

(A-B) Antibody absorption test by LY6G6D recombinant protein pre-incubation using CRC tissue sample. Anti-LY6G6D staining of tumor cells was detected in CRC tissue without pre-incubation of clone 10C1 with recombinant protein (A). No staining was detected in CRC tissue after pre-incubation of clone 10C1 with 50X recombinant protein (B). (C) Representative images of CRC samples with different percentage of LY6G6D stained cells within the tumor area stained with clone clone 10C1 (top), or isotype control (bottom). Bar length is 100 μm.

Supplemental Figure 3

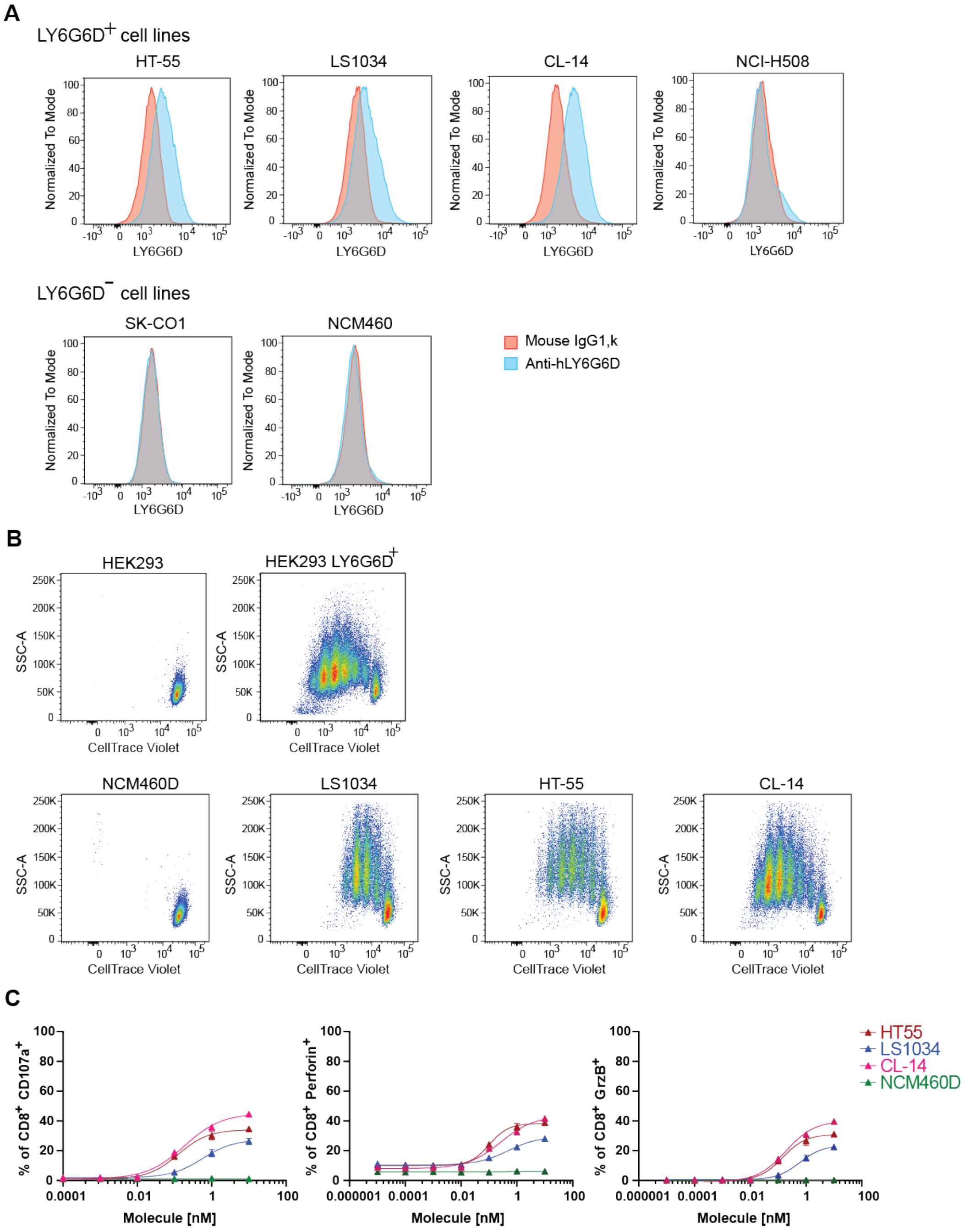

Supplemental Figure 3.

(A) LY6G6D expression on CRC cell lines. (B) LY6G6D-negative (HEK293 and NCM460) and positive (HEK LY6G6D+, LS1034, HT-55 and CL-14) were co-incubated with purified labelled T cells and LY6G6D/CD3 TcE for 5 days. Proliferation of T cells was assessed by dilution of cell tracer. (C) LY6G6D-negative and -positive tumor cells were co-incubated with purified T cells and increasing amounts of concentrations of LY6G6D/CD3 TcE. Degranulation markers were analyzed by FACs after 72 hours of incubation.

Supplemental Figure 4

A

Tissue #	Tumor type	Baseline expression LY6G6D	Baseline expression CD3
1	CRC-MSS	positive	positive
2	CLM	positive	positive
3	CRC-MSS	negative	positive
4	CLM-MSS	positive	positive
5	CLM-MSS	positive	positive
6	CRC-MSS	positive	positive
7	CRC-MSS	negative	positive
8	CRC-MSI H	negative	positive

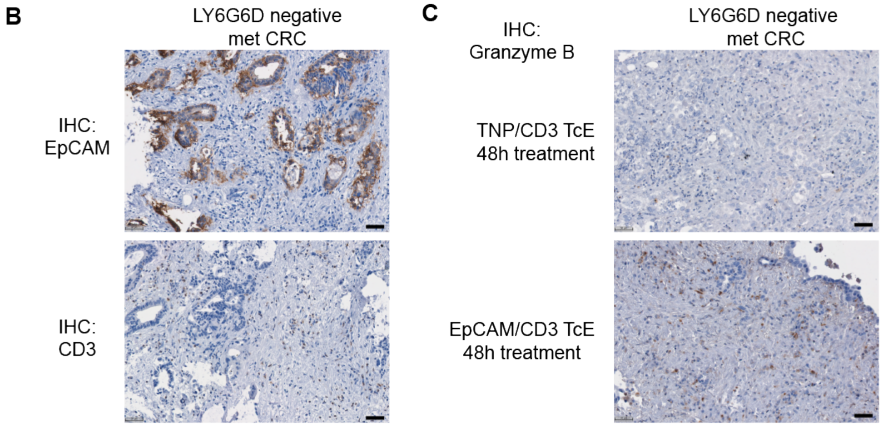

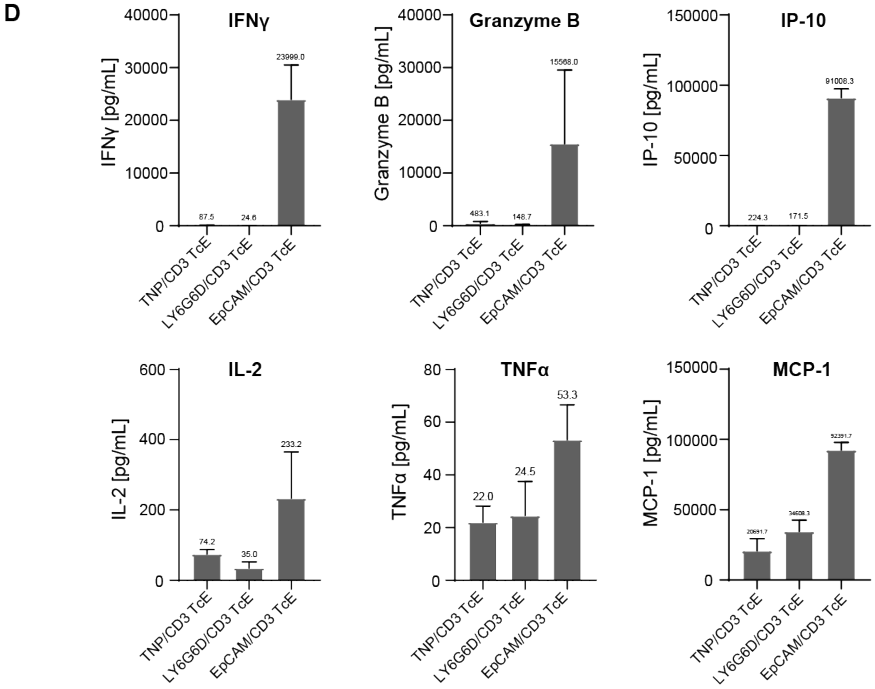

Supplemental Figure 4.

(A) Information on CRC phenotype and baseline expression of LY6G6D and CD3 in CRC tissue samples. (B) EpCAM and CD3 expression in one LY6G6D- CRC tissue sample (tissue #3) at baseline. (C-D) Tissue #3 was cultured for with 1 nM LY6G6D/CD3 TcE, 1nM EpCAM/CD3 TcE or control TNP/CD3 TcE. After 48 hours, Granzyme B staining in the tissue slides (C) and cytokines in the supernatant (D) were assessed.

Supplemental Figure 5

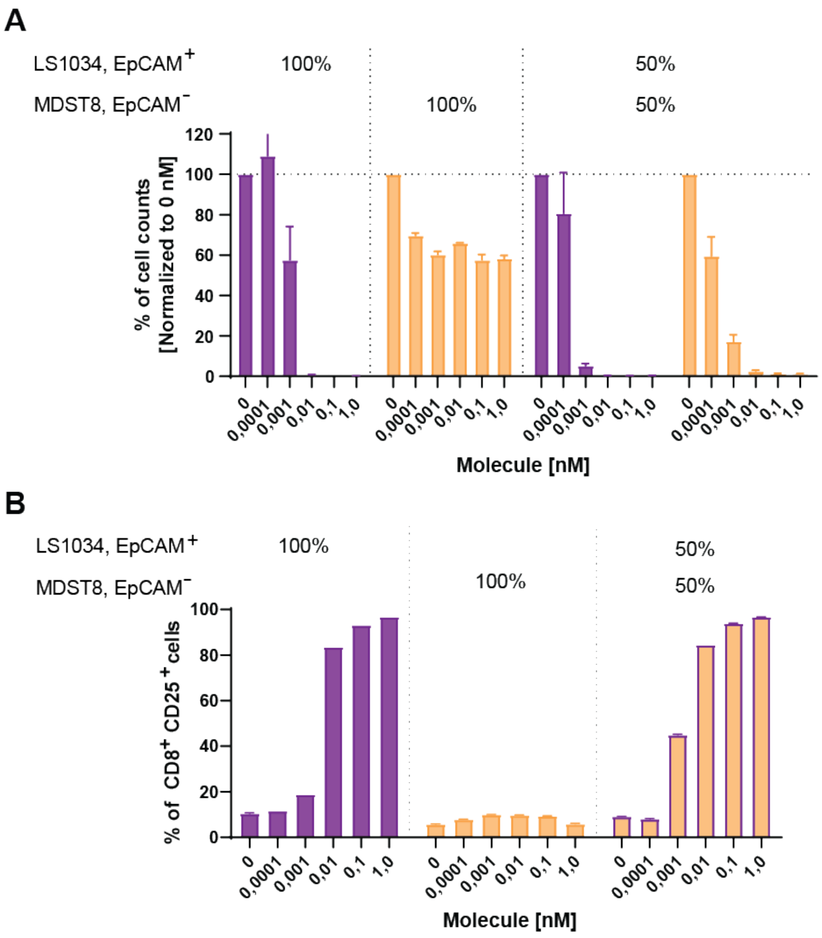

Supplemental Figure 5.

(A-B) EpCAM-positive and -negative (LS1034 and MDST8, respectively) tumor cells were labeled with cell tracer, plated isolated (100%) or co-cultured at 1:1 ratio. Tumor cells were then co-incubated with purified T cells and increasing amounts of concentrations of EpCAM/CD3 TcE. The total number of tumor cells (A) and T cell activation (B) was analyzed after 72 hours of incubation.

Supplemental Figure 6

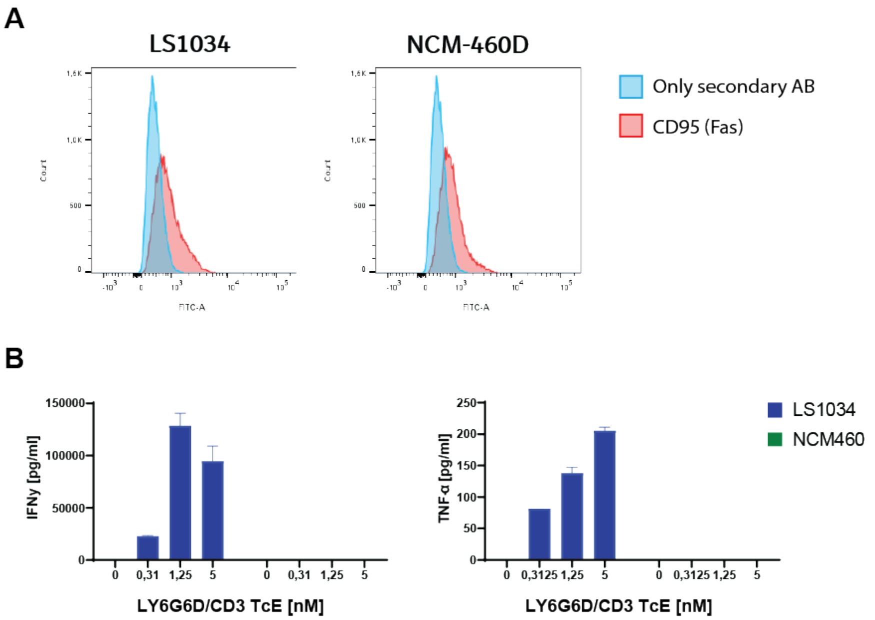

Supplemental Figure 6.

(A) Basal expression of Fas (CD95) in LS1034 and NCM-460D cell lines. (B) LY6G6D-positive (LS1034) or negative (NCM460) tumor cells were co-incubated with purified T cells and increasing amounts of concentrations of LY6G6D/CD3 TcE. IFNγ and TNFα cytokines in the media were assessed after 72 hours of incubation

T cell Proliferation assay:

Target cells were seeded in 96-well plates and pre-incubated at 37°C for 3 hours. Purified T-cells from PBMC (EasySep™ Human T Cell Enrichment Kit; Stemcell Technologies), labelled with CellTrace™ Violet Dye according to manufacturer's instructions. And added in a target to T cell ratio 1:10, along with the LY6G6D/CD3 TcE. After 5 days of incubation T-cells were harvested and stained with Zombie NIR Fixable Viability Dye. T-cells were stained with anti-CD4 and antiCD8 (BioLegend; BD Biosciences) and acquired on FACS Canto-II (BD Biosciences). Analysis of proliferation was performed by FlowJo (TreeStar).

Table of reagents:

Reagent or Resource	Source	Identifier (Catalog #)
**Antibodies**		
Mouse anti-human CD45 (clone 2D1)	BioLegend	368509
Mouse anti-human CD8 (clone RPA-T8)	BD Biosciences	562428
Mouse anti-human CD4 (clone OKT4)	BioLegend	317444; 317414
Mouse anti-human CD25 (clone M-A251)	BioLegend	356110
Anti-human CD25 (clone BC96)	BioLegend	302606
Mouse anti-human CD69 (clone FN50)	BD Biosciences	557745
Human TruStain FcX	BioLegend	422302
Anti-human LAG3 (clone T47-530)	BD Biosciences	745640
Anti-human CTLA4 (clone BNI3)	BioLegend	369604
Anti-human TIM3 (clone 344823)	BD Biosciences	747961
Anti-human PD-1 (clone EH12.2H)	BioLegend	329952
Anti-human FoxP3 (clone PCH101)	eBioscience/ ThermoFisher	17-5773-82
Anti-human Granzyme B (clone 351927)	Invitrogen	MA523639
Anti-human CD107a (clone H4A3)	BioLegend	328641
Anti-human Perforin (clone B-D48)	BioLegend	353312
Mouse anti-human LY6G6D (clone 13.8)	BioLegend	367004
Mouse IgG1kappa (clone MOPC-21)	BioLegend	400114
Anti-LY6G6D	BioLegend	367003
Mouse IgG1 control antibody	Dako	X0931
**Critical Commercial Assays**		
Zombie Green Fixable Viability Dye	BioLegend	423112
Zombie NIR Fixable Viability Dye	BioLegend	423106
CellTrace™ Violet Dye	Invitrogen	C34557
CellTrace™ FarRed	Invitrogen	C34564
CellTrace™ Oregon Green™ 488	ThermoFisher Scientific	C34555
EasySep™ Human T Cell enrichment Kit negative selection	StemCell Technologies	19051
Steady-Glo® Luciferase Assay System	Promega	E2520

Foxp3 / Transcription Factor Staining Buffer Set	eBioscience/ ThermoFisher	00-5523-00
CellTiter 96® AQueous One Solution Cell Proliferation Assay	Promega	G3580
Cytotoxicity detection kit-Plus	Sigma/Roche	04 744 934 001
Cell Line Nucleofector™ Kit V	Amaxa/Lonza	VCA-1003
Phospholipase C Protein, Phosphatidylinositol-Specific	ThermoFisher	P6466
QIFIKIT®	Dako	K0078
CORNING TRANSWELL-96 SYSTEM,.4umPC MEMBRANE	Sigma-Aldrich	CLS3391-5EA
AccuCheck COUNTING beads	invitrogen	PCB100
DMEM-F12, HEPES	Gibco	31330038
Minimum Essential Medium Eagle	Sigma	M5650
RPMI 1640 Medium (ATCC modification)	Gibco	A1049101
Dulbecco's Modified Eagle's Medium – high glucose	Sigma	D6429
M3:baseF™	Incell Inside™	M300F
MEM Non-Essential Amino Acids Solution (100X)	Gibco	11140035
2-Mercaptoethanol	Gibco	21985023
200 mM L-Glutamine	Gibco	25030-024
Trypsin/EDTA	Gibco	043-903174
RPMI-1640 w/o Phenolred	Gibco	32404-014
Fetal Bovine Serum	GE Healthcare	SH30084.03<br>Lot#: DD17630267
Hygromycin B	ThermoFisher Scientific	10687010
Geneticin™ Selective Antibiotic	Gibco	10131035
Incucyte® Caspase-3/7 Dye for Apoptosis	Sartorius	4440
**Recombinant DNA**		
Plasmid: LY6G6D_NFLAG_pcDNA 3.1(+)_A009	Life Technologies	N/A
pGL4.30 NFATluciferase reporter plasmid	Promega	E8481
**Software and Algorithms**		
BD FACSDIVA v	BD Biosciences	
FlowJo V10	FlowJo, LLC	
GraphPadPrism 8.0/9.0	GraphPad Software	
Ensight Software	Perkin Elmer	
SoftMax Pro (ELISA reader)		

Table 1. Cell lines:

Cell line	Source	Culture method
CL-14	Leibniz Institute DSMZ Cat#: ACC 504	DMEM/F-12-20%
HT55	Public Health England<br>cat.nr: 85061105<br>Lot: 09K002	EMEM-20
NCI-H508	ATCC	RPMI-10
LS1034	ATCC-CRL-2158	RPMI-10
NCM460	Incell<br>lot#C2010AUG10-01	M3-base medium-10%
HEK 293	ATCC CRL-1573	EMEM-10
Rec. 293 LY6G6D+	Own description	DMEM-10 + 1 mg/ml G418
MDST8	ECACC<br>Lot: WT13K0001	DMEM-10
SK-CO1	ATCC HTB-39<br>lot: 61834531	EMEM-10
Jurkat NFAT luc	BI Canada, Laval	RPMI-10 (0.25 mg/ml Hygromycin)
